# Effect of Testosterone on Proliferation Markers and Apoptosis in Breasts of Ovariectomized Rats

**DOI:** 10.1055/s-0039-3399552

**Published:** 2019-12

**Authors:** Jussara Celi Conceição Oliveira, Marcelo Luis Steiner, Thérèse Rachell Theodoro, Ana Maria Amaral Antonio Mader, Giuliana Petri, Luiz Carlos Abreu, Maria Aparecida da Silva Pinhal, César Eduardo Fernandes, Luciano Melo Pompei

**Affiliations:** 1 Faculdade de Medicina do ABC, Fundação do ABC, Santo André, São Paulo, Brazil; 2 Department of Gynecology and Obstetrics, Faculdade de Medicina do ABC, Santo André, SP, Brazil; 3 Department of Biochemistry, Faculdade de Medicina do ABC, Santo André, SP, Brazil; 4 Department of Pathology, Faculdade de Medicina do ABC, Santo André, SP, Brazil; 5 Biottery Coordination, Faculdade de Medicina do ABC, Santo André, SP, Brazil; 6 Outline and Scientific Writing Laboratory, Faculdade de Medicina do ABC, Santo André, SP, Brazil

**Keywords:** testosterone, proliferation, apoptosis, breast, ovariectomy, testosterona, proliferação, apoptose, mama, ovariectomia

## Abstract

**Objective** To investigate the action of testosterone (T), isolated or associated with estradiol benzoate (EB), on the proliferation markers and apoptosis of breasts of ovariectomized rats.

**Methods** A total of 48 castrated female Wistar rats were divided into 6 groups, and each of them were submitted to one of the following treatments for 5 weeks: 1) control; 2) EB 50 mcg/day  + T 50 mcg/day; 3) T 50mcg/day; 4) EB 50 mcg + T 300 mcg/day; 5) T 300 mcg/day; and 6) EB 50 mcg/day. After the treatment, the mammary tissue was submitted to a histological analysis and immunoexpression evaluation of proliferation markers (proliferating cell nuclear antigen, PCNA) and apoptosis (caspase-3).

**Results** There was a statistically significant difference among the groups regarding microcalcifications and secretory activity, with higher prevalence in the groups treated with EB. There was no difference among the groups regarding atrophy, but a higher prevalence of atrophy was found in the groups that received T versus those that received EB + T. There was a difference among the groups regarding the PCNA (*p* = 0.028), with higher expression in the group submitted to EB + T 300 mcg/day. Regarding caspase-3, there was no difference among the groups; however, in the group submitted to EB + T 300 mcg/day, the expression was higher than in the isolated T group.

**Conclusion** Isolated T did not have a proliferative effect on the mammary tissue, contrary to EB. Testosterone in combination with EB may or may not decrease the proliferation, depending on the dose of T.

## Resumo

**Objetivo** Investigar a ação da testosterona (T) isolada ou associada ao benzoato de estradiol (EB) na proliferação e apoptose de mamas de ratas ovariectomizadas.

**Métodos** Um total de 48 ratas Wistar castradas foram divididas em 6 grupos, e cada um foi submetido a um dos seguintes tratamentos durante 5 semanas: 1) controle; 2) BE 50 mcg/dia + T 50 mcg/dia; 3) T 50 mcg/dia; 4) BE 50 mcg + T 300 mcg/dia; e) T 300 mcg/dia; e f) BE 50 mcg/dia. Após o tratamento, o tecido mamário foi submetido a análise histológica e avaliação de imunoexpressão de marcadores de proliferação (antígeno nuclear de células proliferantes, PCNA) e apoptose (caspase-3).

**Resultados** Houve diferença estatisticamente significante entre os grupos com relação às microcalcificações e à atividade secretora, com maior prevalência nos grupos tratados com BE. Não houve diferença entre os grupos quanto à atrofia, mas houve maior prevalência de atrofia nos grupos que receberam T *versus* os que receberam BE + T. Houve diferença entre os grupos quanto ao ANCP (*p* = 0,028), com maior expressão no grupo BE + T 300 mcg/dia. Com relação à caspase-3, não houve diferença entre os grupos, mas, no grupo BE + T 300 mcg/dia, a expressão foi maior do que no grupo de T isolada.

**Conclusão** A T isolada não apresentou efeito proliferativo do tecido mamário, contrariamente ao EB. A T em associação ao EB pode diminuir ou não a proliferação, a depender da dose de T.

## Introduction

Testosterone (T) has been used by postmenopausal women, with a positive effect on desire and sexual function.^1^ Through cohort studies in worldwide populations, researchers have reported a small but significant association between T, androstenedione, dehydroepiandrosterone (DHEA) and dehydroepiandrosterone Sulfate (DHEAS), and breast cancer in postmenopausal women.^2^ ^3^ ^4^ Most of the studies using T last from 1 month to 2 years, hence the information that assures its long-term use is unknown, which is a concern for medical professionals as well as for climacteric women regarding the risk and its impact on breast tissue.^5^ ^6^ Thus, considering the increasing use of T treatments by menopausal women, the lack of in vivo controlled studies using T for hormone replacement, and the need for further investigation of its biological impact on the breast tissue, it is relevant to study such aspects in an animal model and the effect of this therapy on the breast. The objective of the present study was to investigate the action of T, isolated or associated with estradiol benzoate (EB), on proliferation markers and apoptosis of mammary tissue in ovariectomized rats.

## Methods

We randomly selected 48 castrated female Wistar rats at 250 days of age. All of them underwent bilateral oophorectomy under anesthesia with ketamine and xylazine intraperitoneally. A longitudinal ventral approach was used for the identification and ligation of the ovarian pedicles and subsequent removal of the gonads. Three weeks later, microscopic examinations of vaginal smears of all rats were performed to confirm hypoestrogenism. Next, the animals were randomly divided into 6 groups of 8 each; the groups received the corresponding daily hormonal dose in a volume of 0.1 ml by subcutaneous injections in the dorsal region of each animal, during 5 consecutive weeks. The daily treatments were as follows: group 1: control, sesame oil 0.1 ml/day (*n* = 8); group 2: EB 50 mcg + T 50 mcg/day (*n* = 8); group 3: T 50 mcg/day (*n* = 8); group 4: EB 50 mcg + T 300 mcg/day (*n* = 8); group 5: T 300 mcg/day (*n* = 8); group 6: EB 50 mcg/day (*n* = 8). The animals were kept in a quiet environment with a constant temperature of 23°C, lighting periods of 12 hours per day, in addition to water and feed ad libitum. Five weeks later, after the end of the treatment, the rats were anesthetized with ketamine 80 mg/kg, and sacrificed by thiopental overdose. The second right mammary gland of each animal was resected and fixed in 10% buffered formalin and subsequently prepared for histological and immunohistochemical evaluation.

### Semiquantitative Histological Analysis

The mammary glands obtained were embedded in paraffin, cut into to 3 μm sections, stained with hematoxylin-eosin (HE), and analyzed under high magnification microscopy (400x). A histological analysis with HE staining was performed, using a semiquantitative method (zero as absent, + as mild, ++ as moderate, and +++ as intense), and the samples were observed using a Nikon (Minato, Tokyo, Japan) double-head microscope. This procedure was performed by the pathologist (A.M.M.) and by the main researcher (J.C.C.O.), and both of them reached a consensus about the presence or absence of atrophy, microcalcification in the glandular lumen, and degree of glandular secretory activity. The quantification of secretory activity was reported as follows:

- +: minimal secretion in the form of cytoplasmic vacuolization without accumulation or dilation of light.
- ++: secretion in the cytoplasm and light, with minimal architectural distortion of the mammary lobes.
- ++ + : exuberant secretion.
- 0: absent secretory activity.

### Histological Analysis: Histomorphometry

The histomorphometric quantitative analysis was performed under HE staining by the pathologist (A.M.M.), using a Zeiss (Berlin, Germany) trinocular microscope with the Axiovision 4 (Zeiss) image analysis software, under medium magnification (100x). The images obtained were captured by a digital camera and transferred to a computer with an image scanner. After calibration to measure in mm^2^, the entire mammary gland (glandular tissue, fibroadipous stroma and striated muscle tissue) was delineated with the computer mouse, obtaining the total area. Then, only the glandular acini were delineated, adding their area, also expressed in mm^2^. Subsequently, the ratio between the glandular area and the total breast area was calculated.

### Immunohistochemistry

Immunohistochemistry (IHC) was performed to analyze proliferating cell nuclear antigen (PCNA) and apoptosis (caspase-3) in 3-μm thick histological sections. All samples were submitted to IHC reaction using the following primary antibodies: PCNA (PC10) 1:1,000 (Cell Signaling Technology, Danvers, MA, US) and caspase-3 1:350 (BioVision, Milpitas, CA, US). The IHC reaction was developed using the peroxidase kit LSAB-peroxidase (DakoCytomation, Copenhagen, Denmark), according to the manufacturer's protocol. Then, they were processed with chromogen (3–3'-diaminobenzidine (DAB) 100 mg (Sigma-Aldrich, St. Louis, MI, US) in 70ml of phosphate-buffered saline (PBS) + 3 ml hydrogen peroxide, and Harris hematoxylin counterstain (Sigma-Aldrich). The slides were analyzed with a Nikon Eclipse TS100 light microscope, under the same light intensity and the same height as the condenser, to identify the areas that best represent the immunostaining of the analyzed molecules (hot spot). The areas that best represented the immunostaining were selected by an experienced pathologist, who was blinded to the treatment groups, and analyzed under 400x magnification . Photomicrographs of consecutive mismatched fields with a resolution of 640 × 480 pixels were obtained using a Nikon Coolpix 4300 digital camera with the same settings for all blades. In each case, the quantification of the immunostaining was performed by digital computer analysis, and the values were expressed by the expression index (IE). The images obtained were analyzed using the ImageLab (Softium Informática, São Paulo, SP, Brazil) image processing and analysis system, adjusted to the micrometer (μm) scale.

## Statistical Method

The data were organized into Microsoft Excel 2007 (Microsoft Corp., Redmond, WA, US) spreadsheets. The qualitative variables were presented as absolute and relative frequencies, and comparisons between the groups were performed using the Chi-squared test.

The numerical data were presented as means and standard deviations, and their normal distribution was analyzed using the Shapiro-Wilk test. As this distribution was not confirmed, the groups were compared using the Kruskal-Wallis test, while comparisons between pairs of groups were made using the Mann-Whitney U-test. The statistical package XLStat (Addinsoft, New York, NY, US), version 17.01, for Excel was used for all analyzes. The level of statistical significance was set at 5% (*p* < 0.05).

### Ethical Aspects

All of the study procedures were approved by the Animal Research Ethics Committee of the Faculdade de Medicina do ABC under number 01/2016.

## Results

During the study there was no loss of animals. After dissection and preparation of the slides, 7 samples had no glandular tissue, so 41 samples with mammary glandular tissue on the slides were included in the analysis.

Table 1 shows the analysis of semiquantitative atrophy parameters, but no statistically significant difference was found among the groups. However, there is a tendency for lower occurrence of atrophy in groups receiving EB and T versus those receiving T alone. Therefore, we decided to group all of the cases that received EB associated with T, as well as those that received T alone, and to perform a comparative analysis of these larger groups, noting a higher prevalence of atrophy in the group treated with isolated T, with statistical significance (*p* = 0.015) (Table 2). As for microcalcifications (Table 1), there was a statistically significant difference among the groups (*p* = 0.024), with a higher occurrence in groups receiving EB. Similarly, we grouped the results of the EB + T groups, comparing them with the groups that received only T (Table 2), and a higher prevalence of microcalcification in the groups that received EB (*p* = 0.022) was observed.

**Table 1:** Results of the semiquantitative parameters by therapy (values expressed in absolute numbers and percentages in parentheses)

Semiquantitative parameters	Placebo n = 8					Estradiol benzoate 50 mcg n = 7
		Estradiol benzoate 50 mcg + testosterone 50 mcg n = 6	Testosterone 50 mcg n = 5	Estradiol benzoate 50 mcg + testosterone 300 mcg n = 8	Testosterone 300 mcg n = 7	
Atrophy^a^						
No	2 (25%)	3 (50%)	0 (0%)	4 (50%)	0 (0%)	2 (28.57%)
Yes	6 (75%)	3 (50%)	5(100%)	4(50%)	7 (100%)	5 (71.42%)
Microcalcification^b^						
Absent	7 (87.5%)	4 (66.6%)	4 (80%)	3 (37.5%)	7 (100%)	2 (28.5%)
Present	1 (12.5%)	2 (33.3%)	1 (20%)	5 (62.5%)	0 (0%)	5 (71.4%)
Secretory activity^c^						
Absent	3 (37.5%)	1 (16.6%)	1 (20%)	1 (12.5%)	6 (85.7%)	0(0%)
Present	5 (62.5%)	5 (83.3%)	4 (80%)	7 (87.5%)	1 (14.2%)	7(100%)
Absent	3 (37.5%)	1 (16.6%)	1(20%)	1 (12.5%)	6 (85.7%)	0(0%)
+	2 (25%)	3 (50%)	4 (80%)	2 (25%)	0 (0%)	2 (28.5%)
+ +	2 (25%)	2 (33.3%)	0 (0%)	3 (37.5%)	1 (14.2%)	3 (42.8%)
+ ++	1 (12.5%)	0 (0%)	0 (0%)	2 (25%)	0 (0%)	2 (28.5%)

Notes: ^a^ *p* = 0.143; ^b^ *p* = 0.024; ^c^ *p* = 0.008. +: mild secretion; ++: moderate secretion; ++ + : intense secretion.

**Table 2:** Results of the semiquantitative parameters (values expressed in absolute numbers and percentages in parentheses) in the groups that received estradiol benzoate plus testosterone and isolated testosterone

Semiquantitative Parameters		
	Estradiol benzoate + testosterone	Testosterone
Atrophy^a^		
No	7 (50%)	0 (0%)
Yes	7 (50%)	12 (100%)
Microcalcification^b^		
Absent	7 (50%)	11 (91.7%)
Present	7 (50%)	1 (8.3%)
Secretory activity^c^		
Absent	2 (14.3%)	7 (58.3%)
Present	12 (85.7%)	5 (41.7%)

Notes: ^a^ *p* = 0.015, ^b^ *p* = 0.022; ^c^ *p* = 0.019.

**Fig. 1:**
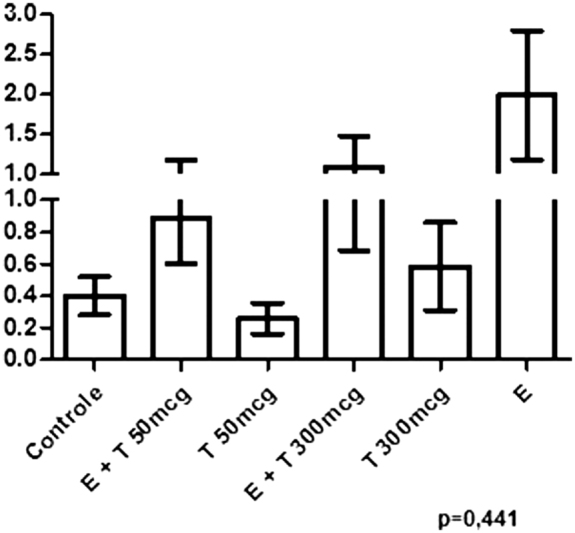
Graphical representation of the mammary gland area in the collected tissue per groups.

The secretory activity was also different among the groups (*p* = 0.008), being higher in the groups that received isolated EB or EB + T compared with the groups that received only T. In Table 2, in the analysis performed with two groups containing all of the cases who received E + T and all of the cases that received isolated T, a higher occurrence of secretion was confirmed in the groups that received EB, with statistical significance (*p* = 0.019). Fig. 1 shows the result of the glandular tissue area. Although the glandular area seemed larger in the groups receiving EB than in those receiving only T or the controls, no statistical significance was found in the overall analysis among groups. In relation to the breast area, no significance was found either.

**Fig. 2:**
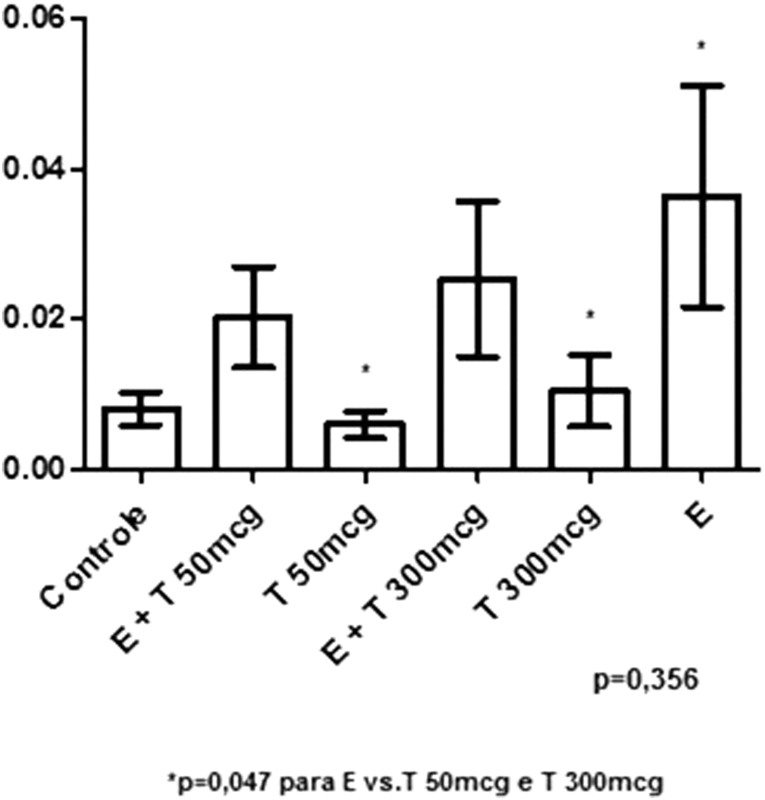
Graphical representation of the ratio between gland area and mammary area per groups.

The graph in Fig. 2 represents the area ratio of mammary acini to the total area of breast, with larger values being observed in the groups treated with EB compared with those treated only with T or the controls, but the overall analysis showed no statistical significance. However, when we compared the cases that received isolated T with the isolated EB group, the difference was statistically significant (*p* = 0.047).

**Fig. 3:**
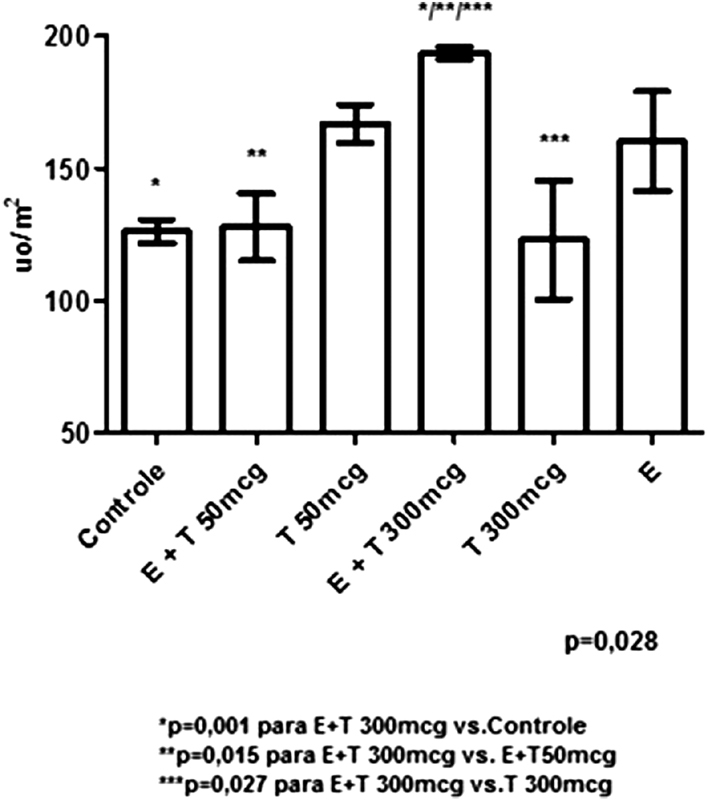
Graphical representation of quantification by immunohistochemistry of PCNA per groups.

The PCNA quantification, shown in Fig. 3, presents a statistically significant difference among groups (*p* = 0.028). The analysis of pairs of groups evidenced a significant difference between the EB 50 mcg + T 300 mcg group and the controls (*p* = 0.001), between the EB 50 mcg + T 300 mcg and EB 50 mcg + T 50 mcg (*p* = 0.015) groups, and between the EB 50 mcg + T 300 mcg and T 300 mcg (*p* = 0.027) groups.

**Fig. 4:**
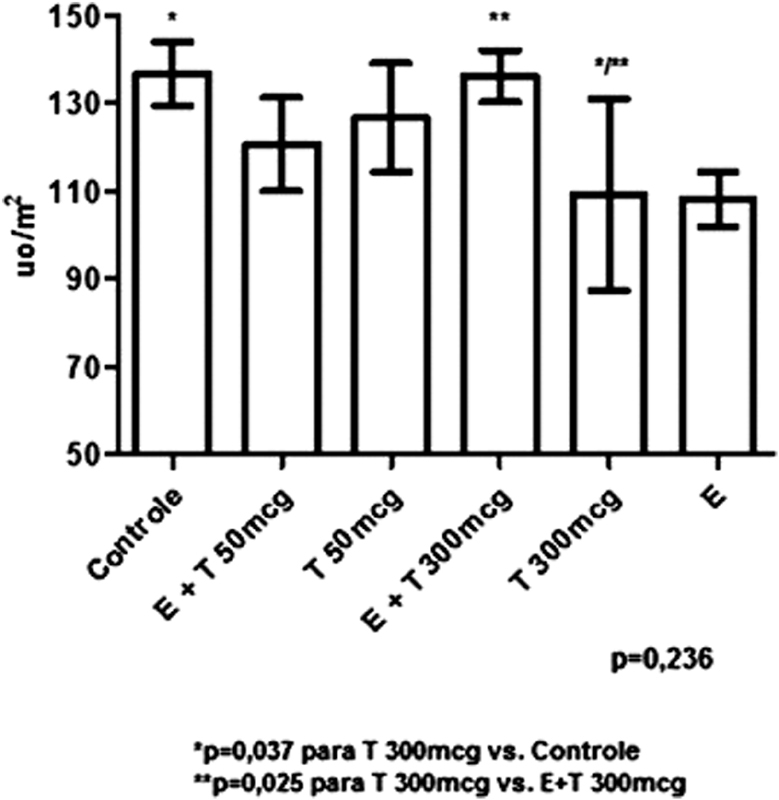
Graphical representation of the quantification by immunohistochemistry of caspase-3 per groups.

Fig. 4 graphically quantifies the immunohistochemistry of caspase-3. No statistically significant difference was found among the groups (*p* = 0.236); however, in the analysis of pairs of groups, a statistically significant difference was observed for the T 300 mcg group compared with the control group (*p* = 0.037), and between the T 300 mcg compared with the E + T 300 mcg group (*p* = 0.025).

## Discussion

Loss of sexual desire, musculoskeletal health impairment, and decreased cognitive performance are some of the characteristic symptoms of the climacteric. The exogenous use of T has been indicated for such complaints, especially for the decrease in sexual desire, but it generates concern among the users. Few studies have been published on the action and effects of T supplementation in women, as well as studies that clarify the contribution of T to the risk of developing cardiovascular, musculoskeletal and cognitive disorders and cancer.^7^

The variety of hormone-related information, specifically T in breast tissue, combined with the divergence of outcomes from studies on the risk of breast cancer and hormone replacement therapy users^8^ ^9^ was sufficient reason to develop this research; however, the difficulties in studying the human breast made us opt for the analysis of the response of mammary tissue to the hormones tested in an experimental model with rats.

The studies by Russo et al^10^ and Russo and Russo^11^ contributed with mammary tissue studies with rats and considered an adequate and representative model of human breast, which supported the decision to choose the murine model in the present study.

The present study did not find a statistically significant difference among the groups for the semiquantitative parameters of atrophy, but when comparing the isolated T groups to the EB + T groups, there was a lower occurrence of atrophy in the groups treated with EB. Thus, we can conclude that, in the absence of estrogen, the mammary lobes atrophy, leading to a decrease in the proliferation of the epithelium, with a decrease in the number of lobes in the breast tissue, as shown by Clarke,^12^ who stated that estrogen is responsible for mitogenesis in the breast during menstruation, even in the presence of T. The novelty is that isolated T in our study did not lead to proliferation; on the contrary the rates of atrophy were higher in the groups treated with isolated T.

The results found in this study are compatible with those of the study by Grynberg et al,^13^ who state that this increase in atrophy of the glandular tissue and fibrosis of the stromal tissues is caused in the postmenopausal period, because of the absence of estrogenic hormonal stimulation in the mammary tissue.

Regarding secretory activity, a statistically significant difference was found in the overall analysis and the analysis among groups, with higher secretory activity in the groups treated with EB + T. This shows that isolated T exerted little stimulus in the proliferative activity of breast tissue, a stimulus that was evidenced whenever EB was added.

Our study showed a statistically significant difference among treatment groups in the proliferative activity measured by PCNA, especially the comparison of groups treated with higher doses of T combined with EB compared with the groups treated with lower doses of T combined with EB, isolated T, or the controls.

Through graphic analysis and based on multiple comparisons, a clear difference in the effect of T/EB on the epithelial proliferation expressed by PCNA is evident. In contrast, a lower rate of proliferation was observed with T alone.

Our study converges with the results of the study by Somboonporn and Davis,^14^ which describes that androgens act in a direct protective way on cell proliferation by controlling the mitogenic effect of estrogen on breast tissue. In addition, one of the few human studies evaluating the effect of T on breast-cell proliferation, conducted by Hofling et al,^15^ found that postmenopausal women who received T (300 mcg/day) associated with hormone-replacement therapy (2 mg of estradiol and 1 mg of norethisterone acetate) showed no increase in cell proliferation; on the other hand, there was a more than five-time increase in cell proliferation in women receiving estrogen therapy associated to progestogen.

This finding suggests that although the histological study has not shown proliferative mammary T activity, there may be a dose-dependent effect when T is associated with EB.

The analysis of caspase-3 showed a higher apoptosis in the group that received EB + T in high doses compared with the group that received the same dose of T, but without EB. This is evidence that the increased proliferation with higher doses of T associated with EB led to increased cell apoptosis.

The present study reinforces the findings of the study by Zhou et al,^16^ who investigated ovariectomized rhesus macaques regarding the sexual steroid effects on mammary epithelial proliferation, with isolated estradiol increasing breast epithelial proliferation by ∼ 50%, and T reducing the induced estradiol proliferation by 40%, suggesting that it is a positive androgen replacement therapy in menopausal women.

Although not always statistically significant, our study shows a lower proliferation in the presence of T in the graphic analysis, but we observed a dose-dependent effect of T, which may mean that, in larger doses, T may not suppress the proliferation induced by estrogen.

Some limitations of our study can be mentioned, such as the fact that we have evaluated proliferation and apoptosis with only one marker for each one, PCNA and caspase-3 respectively, although these have shown results and have already been used in previous studies such as the one by Pompei et al.^17^ Another limiting factor was our relatively small number of animals in the different groups. A larger sample would probably show statistical significance in other parameters that in the graphic analysis seem to be different among groups. As a strong point, the double-blinded design was highlighted, in which the researchers and the pathologist who analyzed the slides were not aware of the treatment in each group, eliminating a source of bias. In addition, comparing different doses of T in combination or not with estrogen helps to understand the possible dose-dependent effect and to support new studies addressing these issues.

## Conclusion

Isolated T did not have a proliferative effect on mammary tissue, contrary to EB. The combination of T and EB may or may not decrease the proliferation, depending on the dose of T.
